# CIB: An Improved Communication Architecture for Real-Time Monitoring of Aerospace Materials, Instruments, and Sensors on the ISS

**DOI:** 10.1155/2013/185769

**Published:** 2013-07-29

**Authors:** Michael J. Krasowski, Norman F. Prokop, Joseph M. Flatico, Lawrence C. Greer, Phillip P. Jenkins, Philip G. Neudeck, Liangyu Chen, Danny C. Spina

**Affiliations:** ^1^NASA Glenn Research Center, 21000 Brookpark Road, Cleveland, OH 44135, USA; ^2^Ohio Aerospace Institute, NASA Glenn Research Center, 21000 Brookpark Road, Cleveland, OH 44135, USA; ^3^U. S. Naval Research Laboratory, 4555 Overlook Avenue SW, Washington, DC 20375, USA; ^4^Jacobs Technology, NASA Glenn Research Center, 21000 Brookpark Road, Cleveland, OH 44135, USA

## Abstract

The Communications Interface Board (CIB) is an improved communications architecture that was demonstrated on the International Space Station (ISS). ISS communication interfaces allowing for real-time telemetry and health monitoring require a significant amount of development. The CIB simplifies the communications interface to the ISS for real-time health monitoring, telemetry, and control of resident sensors or experiments. With a simpler interface available to the telemetry bus, more sensors or experiments may be flown. The CIB accomplishes this by acting as a bridge between the ISS MIL-STD-1553 low-rate telemetry (LRT) bus and the sensors allowing for two-way command and telemetry data transfer. The CIB was designed to be highly reliable and radiation hard for an extended flight in low Earth orbit (LEO) and has been proven with over 40 months of flight operation on the outside of ISS supporting two sets of flight experiments. Since the CIB is currently operating in flight on the ISS, recent results of operations will be provided. Additionally, as a vehicle health monitoring enabling technology, an overview and results from two experiments enabled by the CIB will be provided. Future applications for vehicle health monitoring utilizing the CIB architecture will also be discussed.

## 1. Introduction 

The International Space Station is a unique space vehicle in that it is currently the largest artificial satellite in orbit around the Earth. The U S portion of the ISS has been designated as a national laboratory by the Congress. The ISS provides a unique environment of extreme hot-cold thermal cycling, cosmic radiation exposure [1], atomic oxygen presence, vacuum, and microgravity. This allows for long duration experiments and space testing of devices and structures. While testing and experiments take advantage of this unique environment, facility equipment must operate reliably in it. Electronic components and integrated circuits (IC) are especially susceptible to radiation effects from the environment in LEO. These effects can present themselves in two ways: long-term dose damage associated with total ionizing dose (TID) or through single event effects (SEE). Long-term or TID results in permanently damaging an IC by altering the crystal lattice of the semiconductor, which can result in changing bias voltages and currents which affect circuit operation. Where single event upsets (SEU) are transient, energy is transferred from ionizing particles to the IC. This energy transfer is localized, so individual transistors on an IC are affected. Single events caused by radiation may only result in the flip of a single bit or the corruption of an analog signal. If an affected bit is part of an instruction for microcontroller or processor, the result could result in operational failure. Further, an SEE-induced phenomenon known as a single event latchup (SEL) could also result in a loss of data but, in extreme cases, may cause a hard destructive failure which could result in the permanent loss of a circuit component. With this in mind, care must be taken in the design of any electronic system expected to operate reliably on the ISS for extended period of time and be tolerant of the expected radiation environment.

To support science payloads, the ISS as a facility provides three telemetry communications interfaces [2] for resident experiments with its associated physical layer/protocol: low rate data link: MIL-STD-1553; medium rate data link: Ethernet; and high rate data link: fiber optic. Each interface can provide telemetry to the ground. Increased bandwidth comes with increased cost in development to meet the physical interface requirements. The only interface available throughout the ISS is the MIL-STD-1553 bus. In addition to telemetry data, the Mil-STD-1553 bus performs the command, health, and status data transfer. This is done for safety reasons, so that health and status data are transmitted on the most reliable communication bus. 

The MIL-STD-1553 [3] “Aircraft Internal Time Division Command/Response Multiplex Data Bus” defines a physical layer as well as a bus protocol. The MIL-STD-1553 bus is highly reliable and robust in that it is a deterministic command and response protocol. The ISS adds additional layers to those of the MIL-STD-1553 standard, in effect making the ISS MIL-STD-1553 implementation a superset of the military standard. Which allows ISS MIL-STD-1553 hardware to interface with other space and aircraft platforms, but not necessarily the converse. The MIL-STD-1553 bus is deployed on numerous U S military aircrafts including the F-16 Falcon, F/A-18 Hornet, AH-64 Apache, and P-3C Orion and has also been adopted by North Atlantic Treaty Organization (NATO). The MIL-STD-1553 bus transmits data at 1 Mbit/second. The command and response protocol of the bus adds overhead, reducing the effective bit rate for data transfer. In addition, the ISS adds overhead to the bus transfers. One layer added by ISS to the MIL-STD-1553 telemetry is the Consultative Committee for Space Data Systems (CCSDS) headers, which allow for data telemetry routing in space. Each node in the telemetry system is provided with a unique application identifier (APID), which is part of the CCSDS header, to enable routing. These APIDs allow a user on the ground to receive telemetry and command their node from anywhere with internet connectivity. The ISS allots 12.8 kBytes of telemetry data per second, some of which is used for overhead. The ISS allows commands from the ground to be routed to APIDs residing on the MIL-STD-1553 bus.

Onboard the ISS, there is an Express Logistics Carrier (ELC) facility which is primarily designed to store ISS replacement parts but also has two science payload slots per platform [4]. Each science payload site has 28 V and 120 V power available. A MIL-STD-1553 communications repeater (bus controller) link to the ISS is also provided by the ELC, allowing telemetry data to pass through.

The CIB was needed to be the communications backbone for a permanent testbed on the ELC of the ISS which would utilize the ISS telemetry of the MIL-STD-1553 bus. The CIB needed to provide simpler communication interface to experimenters while maintaining the reliability expected of a space flight system on the ISS. The CIB needed to be designed with radiation tolerance and reliability as primary considerations. The CIB was designed by the NASA Glenn Research Center (GRC) Mobile and Remote Sensing (MaRS) Laboratory. The first experiment set to utilize the testbed was the Materials on the International Space Station Seven (MISSE7) [5]. MISSE7 was comprised of numerous passive experiments and over 21 active experiments using the command and telemetry capabilities provided by the CIB. MISSE7 used two Extra-Vehicle Activity (EVA) deployable suitcase-like containers called a Passive Experiment Container (PEC) in which numerous experiments are contained. These PECs are carried by astronauts, opened, and mounted on the ELC. When the experiment was completed, an additional EVA was used to retrieve the PECs and install a new one for the follow-on experiment set, MISSE8. This exchange required a physical communications link which was swappable. The need for on-orbit exchange of PECs required a physical communications link robust enough to withstand potential damage encountered during the swap.

**Figure 1:**
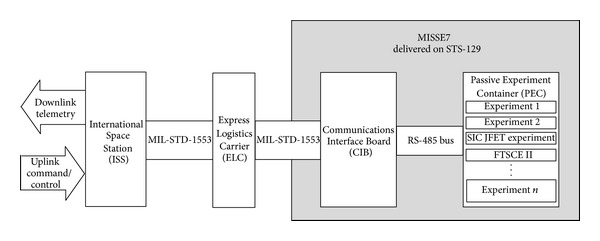
Diagram of the MISSE7 communication architecture and interface to the International Space Station telemetry through the Express Logistics Carrier.

The CIB was installed during Space Transportation System-129 (STS-129) and is currently flying on the exterior of the International Space Station mounted on the Express Logistics Carrier 2 (ELC-2). The CIB provides a simple, RS-485-based communications interface between experiments, instruments, or sensor systems and the ELC's MIL-STD-1553 bus. This allows developers to design on simple platforms without having to confront the difficulty of integrating their experiments directly into the ELC or the ISS. The CIB currently provides serial communication supporting twenty active systems.  Figure 1 shows the CIB within the MISSE7 communication architecture including the interface to the ISS telemetry through the ELC. Follow-on experiments (MISSE8) leave the CIB in place and swap out one or both of the allowed PECs. 

This paper will discuss in detail the technical development and design of the CIB hardware and architecture. Recent results from current operations aboard the ISS will be provided. Specific experiments supported by the CIB like a Silicon Carbide Junction Field Effect Transistor (SiC JFET) health monitoring and a solar cell health monitoring will be discussed. Future health monitoring applications to include node to node schemes will also be briefly discussed. 

## 2. CIB Design

The CIB is designed to be a highly reliable and radiation hard communications bridge from the ISS/ELC MIL-STD-1553 to onboard experiments, sensors, and health monitoring systems. The CIB is constructed from components designed or known to be radiation tolerant of LEO conditions for over 20 years of operation. Further, the CIB is designed to be tolerant of the electrostatic discharge (ESD) anticipated to occur during removal and insertion of new payload systems over multiple missions. 

**Figure 2:**
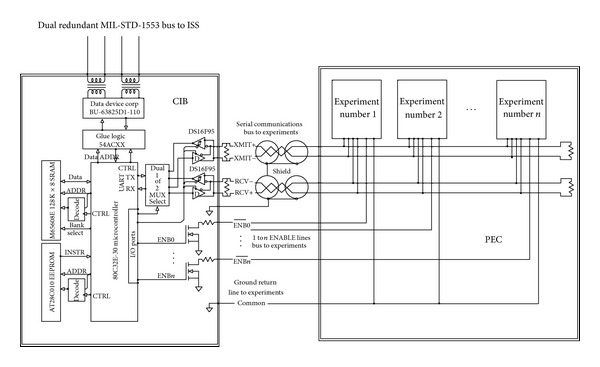
A block diagram of the Communications Interface Board (CIB) interfacing between the International Space Station (ISS) MIL-STD-1553 bus and the experiments residing on the Passive Experiment Container (PEC) (image courtesy of NASA).

Communications over each RS-485 bus was limited to 9600 baud, which is 9600 bits of data per second for eight bit words. Though theoretically capable of at least ten times this bandwidth, the CIB was designed for 9600 baud as a consequence of some experiments in MISSE7 being only capable of that rate due to hardware or software constraints. Thus, the lowest common denominator dictated bus speed. A reprogrammed CIB could support higher baud rates and could dynamically change baud rate to accommodate each experiment. A block diagram of the CIB interfaced to a string of experiment systems is given below in Figure 2. Note that all systems interface to the CIB via a full duplex RS-485 bus system and each system has associated with it a unique hardware enable line.

## 3. Hardware Interface

The RS-485 standard specifies a multidrop serial bus which can be full or half-duplex. The CIB implements a full duplex multidrop bus, where each experiment or sensor is a stub or drop on the bus, and reception and transmission happen on separate lines. Since MISSE7 was to support two physically separate experiment containers, the designers provided two RS-485 buses from the CIB, one for each container. The multidrop bus provides a concern for reliability as one errant transceiver can corrupt the bus. To mitigate against this risk, the CIB implements a hardware transmission enable, as well as requiring a software enable function on the experiment side.

**Figure 3:**
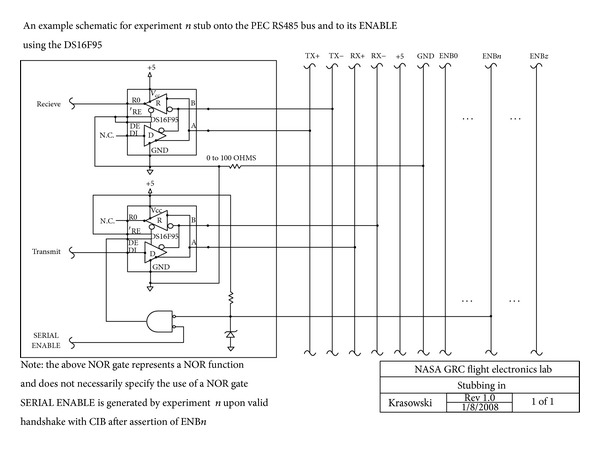
Schematic diagram of the RS-485 bus interface provided to experiments by Communications Interface Board (CIB) (image courtesy of NASA).

Experimenters must provide a hardware handshaking interface as shown in Figure 3, wherein the enable line from the CIB is shown schematically to provide one half of a signal set necessary to enable a system's transmit hardware. The second half of the signal set is provided by the system through response to an initiating packet transfer from the CIB on the RS-485 bus containing the system's APID. This interface prevents experiments from transmitting without being selected and communicated to by the CIB. The interface must be implemented in hardware so that the experiment cannot interfere with the RS-485 bus during communications slots not associated with it. The cause of this out of order bus use could be the result of a software failure, for example. The CIB maintains a permission bitmap that can be overwritten by a command from the ground. If a given experiment is locked out via the bitmap (for any number of scheduling reasons or after detection of system failure), its enable is never asserted and is prevented from communicating.

This transmission scheme allows for the full duplex hardware Universal Asynchronous Receiver Transmitter (UART) common to most microcontrollers/microcomputers/computers or which can be easily configured into programmable logic or as a software UART routine on microcontrollers which allow for interrupt on transition. Thus, systems may be easily configured using simple and available hardware and design tools.

The byte format is 8 bit, one start bit, stop bit, and no parity. The baud rate is 9600 which simplifies requirements for the simpler hardware and offer forgiveness in timing and deskew.

The CIB rotates around through its list of 20 APIDS, polling each experiment one at a time, in order, as long as power is applied. This method is chosen as the desired way to maximize bandwidth for the experiments. If a system needs to pass more than one packet set of data, it merely waits until the CIB returns to it at a later time. 

**Figure 4:**
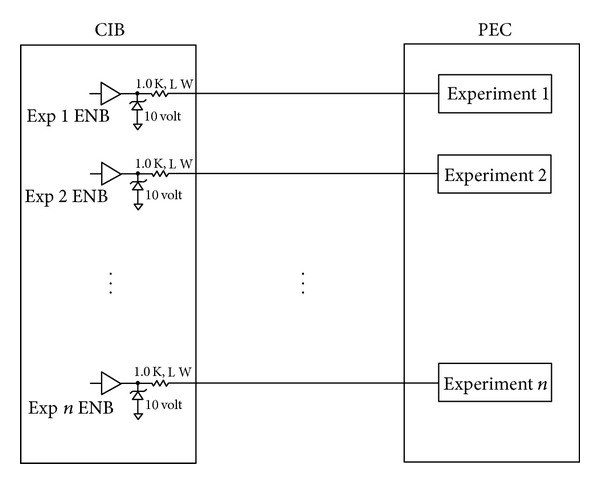
Schematic of the CIB enable lines for experiments onboard the Passive Experiment Containers (PECs). Current limiting resistors as well as voltage limiting Zender diodes to protect CIB enable circuitry from experimental failure are shown within the CIB (image courtesy of NASA).

In addition to the transmission lines (TX), receive lines (RX), +5 Volt power, and ground are the enable lines for the experiments. Within the example experiment hardware is the logic to enable failsafe transmission on the shared RS-485 bus as required by the CIB Interface Control Document [6]. The separate enable wire provided to each experiment is detailed in the following illustration in Figure 4. 

An enable line is pulled low for a particular system prior to the CIB transmitting to that system. A 10 Volt transient absorber (dual redundant 5 volt transient absorbers) and a 1 kΩ resistor occupy the output of each enable line as shown in Figure 4 and are present to mitigate against electrostatic discharge and also to a short to +28 condition should it occur at an experiment. As noted earlier, the enable wire is used by the system to enable its transmitter hardware if it has also received a valid packet from the CIB. Commencement of the packet transfer from CIB occurs no less than 250 ms from the assertion of enable. This enable signal removes the obligation of the systems from having to monitor all the traffic on the TX line while listening for transmissions dedicated to themselves. 

Also, this active low enable signal can be used to locally enable a pass element to provide the system power. Thus, an experiment may come alive upon a powerup initiated by this signal. The systems are expected to be capable of accepting a packet from the CIB 250 milliseconds from the assertion of the enable, and so boot-up must occur consistent with this delay. Failure to receive this command and reply after 250 ms results in the CIB disasserting the enable line and moving on to the next experiment. This is of consequence to a system which uses the time between sequential acquisitions of itself by the CIB to perform its tasks. After completion of task and data transfer with the CIB, a system may turn itself off. Removal of power when not operating reduces some radiation total dose effects and can also remove the conditions for and thus clear a nondestructive latchup.

Each transfer starts with a packet from the CIB containing the experiments APID, and so theoretically experiments do not need to consider the state of an enable line but merely listen for their APID. Given this fact and since the two communications busses are RS-485 links, with proper wiring considerations, over thirty experiments per RS-485 bus are possible with a reprogrammed CIB.

## 4. Software Description

 The CIB firmware is built on a commercially available real-time kernel. The firmware consists of serial input/output, CCSDS packet building, time keeping, and MIL-STD-1553 support modules in addition to top-level modules that manage data handling and poll experiments. 

 Data handling is straightforward. Experimenters have only to implement a very simple protocol to enable communication. All packets have a fixed length. Command packets are 116 bytes long, data packets are 1274 bytes long, and acknowledgment packets are 10 bytes long [6]. The CIB will query experiments one at a time. Each query has the form in Box 1.

The firmware packs experiment data into CCSDS packets for transmission and unpacks commands received from the ground. This relieves developers from the task of implementing CCSDS packet handling.

 Experiments can be controlled through commands of up to 104 bytes in length. No processing is done on commands by the CIB; they are simply passed to the experiment. Commands may contain any kind of data including firmware updates. Experiments are polled in round-robin fashion, each transaction requiring roughly 2.5 seconds. Experiments can transmit up to 2 MB/day. An experiment may receive a command from the ground or transmit data during a single transaction. Data from the experiment is wrapped in a CCSDS packet and buffered in the MIL-STD-1553 transceiver.

 MIL-STD-1553 transactions occur in frames. The minor frames contain different transactions. A major frame consists of minor frames. The major frames are repeated. The ELC uses ten 100 ms minor frames for each major frame. The result is that data packets received from experiments are buffered a maximum of one second before being transmitted on the MIL-STD-1553 bus. In addition to passing data, the MIL-STD-1553module initializes the transceiver, updates the time, and transmits the health and status packets of Table 1 once per major frame.

## 5. CIB Hardware

The core of the CIB is the 80C32E-5962-0051801QQC radiation tolerant 8 Bit ROMless microcontroller. This performs all processing functions within the system. The 80C32E has the following environmental operating specifications [7]: temperature range is military (−55°C to 125°C), no single event latch-up (SEL) below a linear energy transfer (LET) threshold of 80 MeV/mg/cm^2^, and is tested up to a total dose of 30 krads (Si) according to MIL-STD-883 method 1019.

Program memory for the CIB is stored in the AT28C010-12DK-MQ-128Kx8 parallel EEPROM which is reprogrammable in circuit at the bench but which is not reprogrammable on-orbit. The AT28C010 has the following environmental operating specifications [8]: temperature range is military (−55°C to 125°C), no SEL below an LET threshold of 80 MeV/mg/cm^2^, and is tested up to a total cose of (according to MIL-STD-883 method 1019): 10 krad (Si) read-only mode when biased and 30 krad (Si) read-only mode when unbiased.

Dynamic memory for the CIB is embodied in an M65608E-5962-8959818MZC radiation tolerant 128Kx8 very low-power Complementary Metal Oxide Semiconductor Static Random Access Memory (CMOS SRAM). The M65608M has the following environmental operating specifications [9]: military temperature range is (−55°C to +125°C), no SEL below a LET threshold of 80 MeV/mg/cm^2^ @ 125°C, and is tested up to a total dose of 30 krad (Si) according to MIL-STD-883 method 1019.

MIL-STD-1553 communications are effected through the BU-63825D1-300 Space Advanced Communication Engine (Sp'ACE II) BC/RT/MT interface module. The Sp'ACE II has the following environmental operating specifications [10]: total gamma dose immunity of 1 × 10^6^ Rad, LET threshold of 63 MeV/mg/cm^2^, and a soft error rate of 2.56 × 10^−5^ errors/device-day.

The core components of the CIB hardware are listed in Table 2 along with their relevant environmental specifications.

**Figure 5:**
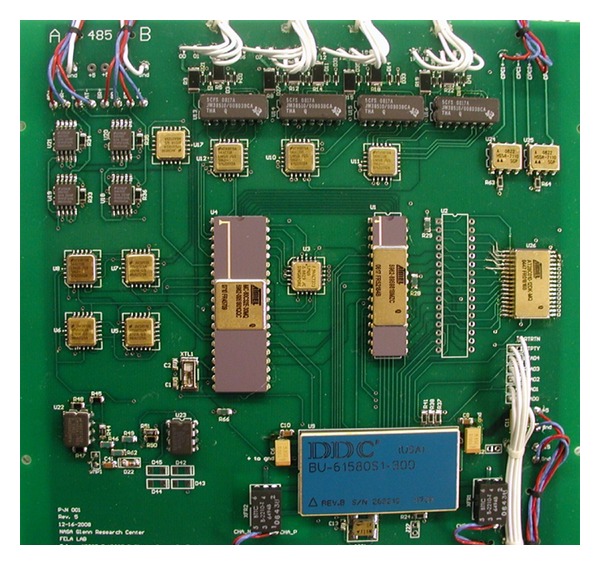
Photograph of the flight Communications Interface Board (CIB) circuit board. This image was taken prior to delivery, during functional testing of the circuit board, and prior to the insertion of the flight MIL-STD-1553 transceiver (image courtesy of NASA).

MIL-STD-1553 coupling transformers and the two clock crystals are screened for space applications. All other active components are deemed radiation hard to this mission through consultation with the customer. A photograph of the CIB is given in Figure 5. The radiation environment in LEO consists primarily of trapped protons with some galactic cosmic rays and solar particles. The expected and agreed to limit for total ionizing dose for the CIB was less than 300 rad(si) per year (behind shielding). The CIB componentry is accepted to be SEU tolerant and SEL hard to the ISS LEO radiation environment. As such, the CIB was designed for long duration survival to the LEO radiation environment.

## 6. Results

The Communications Interface Board, or CIB, embodies the first demonstration of a permanent communications and control interface for deployable experiments to characterize materials, systems, and components exposed to LEO. The CIB was and is the core of two of the earliest science payloads on the ELC affixed to the external structure of the ISS as part of an Express Payload Adapter (ExPA). These two experiments, deployed by astronauts during EVA, are MISSE7 and -8. MISSE7 was deployed, had a successful mission, and was subsequently returned to Earth. MISSE8 remains on orbit at the time of this writing. The CIB was delivered to the ISS on STS-129 in November 2009 along with MISSE7.  Table 3 lists experiments which use command and telemetry data provided by the CIB. MISSE7 then returned when MISSE8 was delivered in May 2011, with the CIB remaining aboard to provide the simplified telemetry interface for MISSE8.  Table 4 lists MISSE8 experiments utilizing command and telemetry data enabled by the CIB. MISSE8 is scheduled for return in March 2014.

The original intent for the CIB was to embody the communications component of a MISSE-specific infrastructure capable of supporting two PECs with up to 20 separate experiments with power, uplink, and downlink capabilities. A PEC is a suitcase-like metal container which, when opened and deployed, presents two opposing experiment surfaces. Two PECs can thus provide zenith and nadir along with ram and wake presentations. This infrastructure greatly reduces the ISS interface complexity for future MISSE experiments. Experimenters would thus have a well-defined power and communication interface to the ISS to build to as well as an EVA compatible mechanical structure for deploying two MISSE style PECs. Further, continuous monitoring of components and samples while on orbit removes the requirement of an experiment returning to Earth for postmission analysis. As such, at end of mission, experiments may be disposed of via deorbiting as will be the case for part of MISSE8. 

 The decommissioning of the space shuttle brought with it a loss of EVA deployable experiments, and as such the PEC structure is inconsistent with new requirements for robotic deployment of science payloads and environmental sensors for insertion onto the ELCs. However, the CIB arguably represents a communication and control interface for a system consistent with current specifications. 

While the CIB hardware currently residing on ELC2 onboard the ISS may not be utilized after the completion of MISSE8, it should be recognized that the architecture is suitable for future use in vehicle health monitoring applications. The current hardware design has been proven to be highly reliable in the demanding space flight environment. The simple telemetry interface provided by the CIB enabled the experiments listed in Table 3 on MISSE7 and Table 4 on MISSE8, respectively. Broad applications enabled by the CIB should be noted, from processor testing to solar cell health monitoring and from CMOS image sensor testing to a variety of materials testing. Two individual experiments specifically related to vehicle health monitoring will be discussed in the next section.

## 7. Health Monitoring Enabled by the CIB

### 7.1. SiC JFET Health Monitoring Experiment

An example of a health monitoring circuit flown on MISSE7 was the silicon carbide (SiC) Junction Field Effect Transistor (JFET) Experiment designed by the NASA GRC mobile and remote sensing laboratory and SiC development group. NASA GRC has a long history in extreme temperature range silicon carbide electronics and packaging development having demonstrated SiC logic circuits operating over a temperature range of −125°C to 500°C [11]. Current long duration extreme temperature testing is performed in laboratory ovens and cold chambers, but future use is anticipated on flight vehicles. In an effort to demonstrate the technology in a flight environment, a health monitoring experiment was designed for SiC JFETs in high temperature packaging which was the first space flight of this technology. The experiment consisted of two SiC JFETs, one in room temperature commercial packaging, the other in high temperature packaging developed by the Ohio Aerospace Institute and NASA GRC [12].

The experiment monitors the current versus voltage transfer characteristics or a curve trace of both transistors during the flight. The transfer characteristics of the transistors show any electrical or physical degradation of the transistors, which is the primary concern of this experiment demonstration. This transfer characteristics are generated with a microcontroller-based curve tracing circuit. The CIB RS-485 protocol includes a timestamp in each transaction, which the SiC JFET uses to determine when to initiate a curve trace. To minimize bandwidth used, the SiC JFET experiment will only run once every hour. When the CIB queries this experiment, if the timestamp does not lie in the first ten minutes of the hour, the experiment will power itself down to wait for the next query. In addition to using the CIB enable line as a safety to lockout transmission on the RS-485 bus, this experiment uses the enable line as a signal to a latch to enable powering up. The CIB assertion of the enable line powers the experiment up, after which the experiment listens for the CIB command packet. The experiment receives the timestamp, determines if the time is within the ten-minute window, and then either proceeds with curve tracing or goes to sleep.

**Figure 6:**
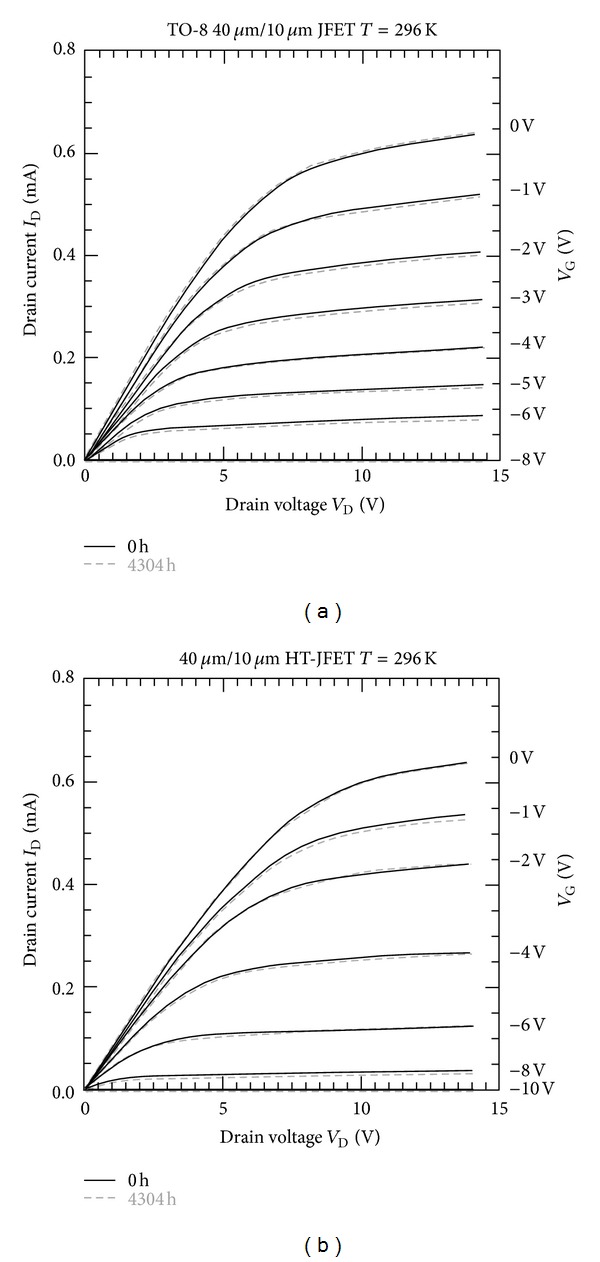
Current versus voltage transfer curves for given gate voltages of the two flight silicon carbide (SiC) Junction Field Effect Transistors (JFETs). (a) shows characteristics for room temperature packaged JFETs, for preflight, and after over six months of flight. (b) shows the characteristics for the high temperature packaged SiC JFET (image courtesy of NASA).

Figure 6 shows experiment data in the form of curve traces from midflight monitoring of the SiC transistor health. Both the room (Figure 6(a)) and high temperature (Figure 6(b)) packaged transistor test data are shown. The graph overlays preflight curves with those 6 months into the flight. These midflight results show no degradation of transistor performance during the 6-month period [12].

**Figure 7:**
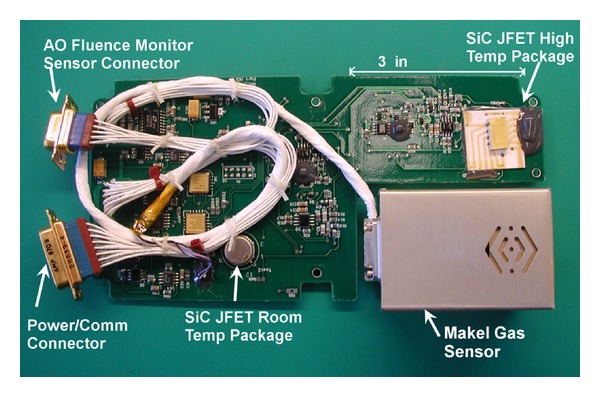
Silicon carbide junction field effect transistor (SiC JFET) health monitoring flight experiment circuit board (image courtesy of NASA).

The experiment board shown in Figure 7 resided at APID 1301 as shown in Table 3. In addition to the SiC JFET experiment, the circuit board and electronics set also supported two additional experiments the Makel Gas Sensor and the atomic oxygen fluence monitor, neither of these will be discussed. The design of the CIB enables many types of health monitoring to be performed, with the sample rate being the limiting factor.

### 7.2. Forward Technology Solar Cell Experiment II

Another experiment toward vehicle health monitoring enabled by the CIB is the Forward Technology Solar Cell Experiment II (FTSCE II). The experiment was designed by the U. S. Naval Research Laboratory with instrumentation development by the NASA GRC MaRS Lab. This experiment monitored solar cell health by measuring current versus voltage (I-V) curve characteristics. Space vehicles in Earth orbit rely on photovoltaic or solar cells as their power source. Space environment effects can degrade photovoltaic cell performance providing less power to the vehicle, and thus affecting the health and operating envelope of the space vehicle. While technology can be used to mitigate the degradation, the space vehicle designer and operator should know how the system will degrade and respond to the changing power system output.

**Figure 8:**
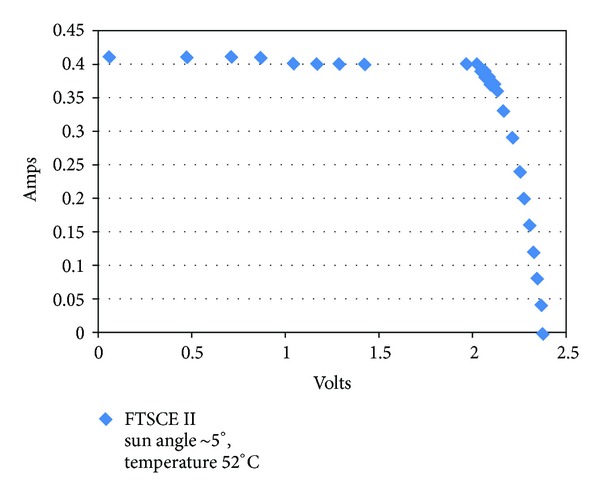
Typical I-V (current versus voltage) curve for a solar cell as measured by the Forward Technology Solar Cell Experiment II (FTSCE II).

**Figure 9:**
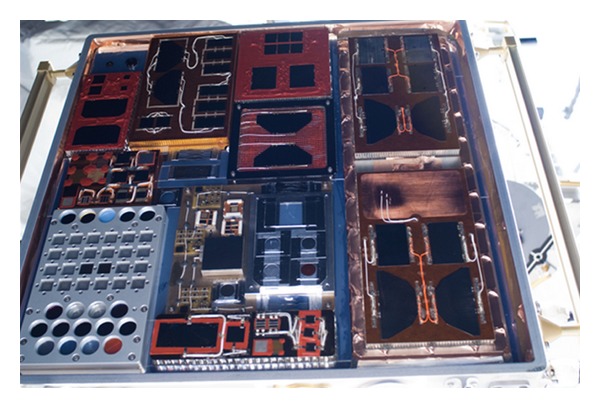
Sun looking face of the Passive Experiment Container (PEC) containing passive material samples and cells as flown on Materials International Space Station Experiment 7 (MISSE7). Solar cells can be denoted by the wires to attach to the measurement circuitry of the Forward Technology Solar Cell Experiment II (FTSCE II) (image courtesy of NASA).

Photovoltaic cells have current versus voltage characteristic which can provide insight into the health of the cell.  Figure 8 shows an example of a solar cell I-V curve from initial FTSCE II on-orbit data. Performance degradation of the cell will show up as less current output, shifting the curve in Figure 8 downward along the vertical axis as the cell degrades. FTSCE II is comprised of 36 experimental solar cells as shown in Figure 9 along with measurement circuitry to provide I-V curve data along with environment parameters of temperature and sun position in relation to the solar cells. The measurement hardware of FTSCE II flown on MISSE7 is actually a reflight of the same hardware originally designed for and flown as FTSCE I on MISSE 5 [13]. FTSCE II resided at the APID 1306 and would transmit data packets consisting of I-V curve data along with relevant environmental parameters. Future results covering the complete FTSCE II experiment are expected, while selected results from in flight data are presented in [14–17]. 

## 8. Future Health Monitoring Applications 

The CIB design discussed in this paper is one possible hardware implementation of the architecture. This design was focused on providing reliable operation within a radiation environment. So, the hardware implementation described represents a design to achieve this goal. The architecture of the CIB may also be realized with a different hardware set to suit other needs. For instance, in a more benign environment, the choices for suitable hardware increase significantly and the design could also be shrunk physically. The hardware could be implemented in a variety of processing platforms which might even include the MIL-STD-1553 interface onboard. One likely hardware target for the CIB architecture is a field programmable gate array (FPGA). This reconfigurable platform would allow the processing unit and communication to exist in single IC, greatly shrinking the physical footprint of the circuitry. A smaller CIB would provide greater access to additional flight platforms.

Another consideration for future use is the reliability of the sensors and instruments which utilize the RS-485 bus. As the sensors mature and are demonstrated to be more reliable in operation, the enable lines provided by the CIB might not be needed. This elimination would reduce the wiring needed as well as the physical size of the CIB.

Hardware changes are not the only possible architecture modifications which would lead to expanded use. Software alterations may also be applied to allow increased use of the architecture. For instance, current CIB protocol might be expanded. The CIB operated with a command/response protocol, where the CIB controlled the RS-485 bus in much the same way as the bus controller in the MIL-STD-1553 bus. One feature possible on a command/response bus is node to node communication. Because the CIB uses packet-based transfers, the software protocol could be expanded to include these node to node transfers, whereby a request for node to node transfer is communicated to the bus controller, which is then granted by the CIB through an additional message. Even in the current configuration, node to node transfer is possible, since a node needs only to actively listen to the bus, for messages encoded in its packet.

Future use of this architecture may be a vehicle health monitoring bus included in aircraft/spacecraft electronic systems. The CIB or future hardware could be the interface between all health monitoring sensors and the crafts central control unit. Used in this manner, the CIB could provide the craft with vehicle health information through a single interface such as the MIL-STD-1553 bus.

## 9. Conclusion

This paper demonstrates that the CIB is an improved architecture enabling simple vehicle health monitoring on the ISS. While the ISS has in place a communications architecture which enables health monitoring, telemetry, and control, the CIB improves and adds to this infrastructure by providing a simpler interface. This is accomplished by interfacing with the ISS MIL-STD-1553 bus and providing two multidrop RS-485 busses for experiments, sensors, and health monitoring systems to communicate on. The RS-485 busses make available the MIL-STD-1553 telemetry, command, and control services without the complex development required for MIL-STD-1553. This simpler interface enables sensor and instrumentation development for vehicle health monitoring applications.

The CIB provides this simpler communication interface while maintaining the reliability expected of a space flight system on the ISS. The CIB was developed with radiation tolerance and reliability as the primary design considerations. With over three years of successful operation on the ISS over two missions, the CIB has proven to be highly reliable and tolerant to the LEO radiation environment while providing simpler vehicle health monitoring communication support.

The CIB also enabled future materials and device development which lead to further use in health monitoring systems. This paper gave two examples of a health monitoring experiment flown on the external of the ISS for over a year. The SiC JFET is a high temperature component which has uses throughout a vehicle to include health monitoring in extremely hot environments. The flight on MISSE7 was the first space flight of this technology and was only possible with the simple telemetry interface provided by the CIB. FTSCE II demonstrated solar cell health monitoring on the ISS with real-time telemetry enabled by the CIB. 

The CIB design discussed is one implementation of the architecture. This architecture might be expanded on or implemented differently for a different platform. Another processing technology might be utilized in the architecture in a more benign environment such as an aircraft. Future software development may use the CIB protocol to support node to node transfer, allowing sensor fusion techniques for vehicle health monitoring.

## Figures and Tables

**Box 1:**
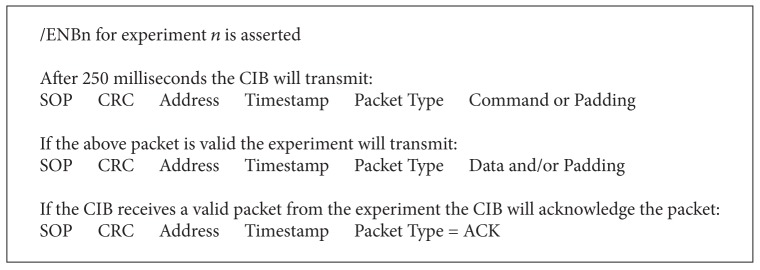


**Table 1:** Health and status packet format transmitted once per major frame (once per second).

Type	Variable	Description
Unsigned char	opto[2]	State of optoisolators that determine whether the AO and FTSCE experiments are enabled
Unsigned long	last_poll_time	Beginning of the current polling cycle
Unsigned short	poll_cycles	Number of completed polling cycles
Unsigned char	poll_map[20]	Determines which experiments are to be polled
Unsigned short	sequence[20]	Packet sequence number of each of the experiments
Unsigned char	valid_crc[20]	0×FF = valid
Unsigned long	last_cmd_time[20]	Time last experiment command received
Unsigned long	last_cib_cmd_time	Time last CIB command received
Unsigned char	valid_ack[20]	0×FF = valid
Unsigned short	cmd_cnt[20]	Count of commands sent to experiments
Unsigned short	cib_cmd_cnt	Count of commands sent to CIB
Unsigned short	err_cmd	Command for nonpolled APID. Error = APID. 0×FFFF = no error
Unsigned short	err_poll	Invalid polling table received. Unused
Unsigned char	last_cmd[106]	Last command transmitted to experiments
Unsigned char	last_cib_cmd[106]	Last command transmitted to the CIB
Unsigned char	last_ftsce_cmd[100]	Last command sent to FTSCE
Unsigned char	ftsce_data[124]	Data transmitted by FTSCE
Unsigned char	reserved[64]	

**Table 2:** CIB core system components and respective environmental operating specifications.

Function	Part number	Temperature range	Single event latchup (SEL) threshold	Total radiation dose
Microcontroller	80C32E	−55°C to 125°C	>80 MeV/mg/cm^2^	30 krad (Si)
Program memory	AT28C010	−55°C to 125°C	>80 MeV/mg/cm^2^	10 krad (Si) under bias 30 krad (Si) when unbiased
Dynamic memory	M65608E	−55°C to 125°C	>80 MeV/mg/cm^2^	30 krad (Si)
MIL-STD-1553 interface module	BU-63825		Immune	1 Mrad

**Table 3:** Materials on the International Space Station Experiment Seven (MISSE7) flown on ISS from November 2009 to May 2011.

Short experiment name	APID	Experiment description	Provider
MCPE	1300	Multicore processor single event upset testing	Naval Research Laboratory
GRC experiment set	1301	SiC transistor health testing [12], H2 sensor, and zenith/nadir AO monitor	NASA GRC
SEUXSE	1302	Xilinx FPGA SEU Testing	Sandia National Laboratory
Not used	1303		
Not used	1304		
CIE	1305	CMOS imager experiment	Assurance technology
FTSCE II	1306	Solar cell health monitoring [14–18]	NRL, NASA GRC, and AFRL
Boeing ram side experiment	1307	Materials testing	Boeing
HyperX	1308	High performance low-power processor, SEU testing [19]	NASA GSFC
SpaceCube “A”	1309	Advanced processor design SEU testing [20, 21]	NASA GSFC
SpaceCube “B”	1310	Advanced processor design SEU testing [20, 21]	NASA GSFC
Wake AO fluence monitor	1311	Wake atomic oxygen fluence monitor/thermal control paints Experiment	NASA GRC
Boeing wake side experiment	1312	Materials testing	Boeing
AFRL wake 1	1313	Tribology measurements	AFRL Dayton, U of Florida
AFRL wake 2	1314	Tribology measurements	AFRL Dayton, U of Florida
AFRL ram 1	1315	Tribology measurements	AFRL Dayton, U of Florida
AFRL ram 2	1316	Tribology measurements	AFRL Dayton, U of Florida
iMESA	1317	Miniaturized electrostatic analyzer [22]	U.S. Air Force Academy
LTESE	1318	Lead-free technology experiment in space environment	MSFC
Ames	1319	Thermal protection systems sensors	NASA ARC, NASA LaRC, NASA JSC, and Boeing
Boeing PICA	1320	Materials testing	Boeing
Ram atomic oxygen fluence monitor/thermal control paints experiment	1321		NASA GRC

**Table 4:** Materials on the International Space Station Experiment Eight (MISSE8) flown on ISS from May 2011 to present (April 2013).

Short experiment name	APID	Experiment description	Provider
None	1300		
Reflectarray	1301	Characterize performance of components of a flexible, phased array antenna	NASA GRC
SEUXSE	1302	Xilinx FPGA SEU testing	Sandia National Lab
Not used	1303		
Not used	1304		
PASCAL	1305	Primary arcing effects on solar cells at LEO	Lockheed Martin, JAXA, Kyushu Institute of Technology
FTSCE III	1306	Solar cell health monitoring [23–25]	NRL, NASA GRC, and AFRL
Not used	1307		
HyperX	1308	High performance low-power processor, SEU testing [19]	NASA GSFC
SpaceCube “A”	1309	Advanced processor design SEU testing	NASA GSFC
SpaceCube “B”	1310	Advanced processor design SEU testing	NASA GSFC
